# Stemofoline ethyl acetate solvate

**DOI:** 10.1107/S1600536809026889

**Published:** 2009-07-18

**Authors:** Pitchaya Mungkornasawakul, Stephen G. Pyne, Alison T. Ung, Araya Jatisatienr, Anthony C. Willis

**Affiliations:** a School of Chemistry, University of Wollongong, Wollongong, NSW 2522, Australia; b Department of Chemistry and Forensic Science, University of Technology Sydney, PO Box 123, Broadway, Sydney, NSW 2007, Australia; c Department of Biology, Chang Mai University, Chang Mai 50202, Thailand; d Research School of Chemistry, The Australian National University, Canberra, ACT 0200, Australia

## Abstract

Crystals of the title compound, C_22_H_29_NO_5_·C_4_H_8_O_2_, {[systematic name: (2*R*,3*R*,5*R*,5a*S*,6*R*,8a*R*,9*S*)-(5*Z*)-5-[3-butyl­tetra­hydro-6-methyl-2,5-methano-4,3,8a-[1]propan­yl[3]yl­idene­furo[3,2-*f*][1,4]oxazepin-7(5*H*)-yl­idene]-4-meth­oxy-3-methyl­furan-2(5*H*)-one ethyl acetate solvate} were isolated from the root extracts of *Stemona aphylla* (Stemonaceae). The structure closely resembles those of stemofoline derivatives which have previously been reported. Inter­molecular contacts are observed between some C-bonded H atoms and nearby O atoms, perhaps indicating weak inter­actions which could influence the packing of species within the unit cell.

## Related literature

For the single-crystal X-ray structure and absolute configuration of stemofoline as the hydro­bromide monohydrate, see: Irie *et al.* (1970 ▶). For two stemofoline alkaloids with structural modifications in the butyl side chain, see: Seger *et al.* (2004 ▶). For the isolation of stemofoline from the root extracts of *Stemona aphylla* (Stemonaceae), see: Mungkornasawakul *et al.* (2009 ▶). For details of the weighting scheme used, see: Watkin (1994 ▶); Prince (1982 ▶).
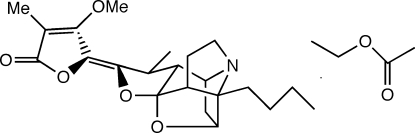


## Experimental

### 

#### Crystal data

C_22_H_29_NO_5_·C_4_H_8_O_2_
*M* *_r_* = 475.58
Orthorhombic, 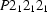

*a* = 10.3908 (1) Å
*b* = 10.6549 (2) Å
*c* = 22.4143 (4) Å
*V* = 2481.55 (7) Å^3^
*Z* = 4
Mo *K*α radiation
μ = 0.09 mm^−1^
*T* = 200 K
0.45 × 0.08 × 0.06 mm

#### Data collection

Nonius KappaCCD diffractometer
Absorption correction: integration via Gaussian method (Coppens, 1970 ▶) implemented in *maXus* (Mackay *et al.*, 2000 ▶) *T*_min_ = 0.976, *T*_max_ = 0.996
41745 measured reflections
3209 independent reflections
2149 reflections with *I* > 2σ(*I*)
*R*_int_ = 0.060

#### Refinement

*R*[*F* ^2^ > 2σ(*F* ^2^)] = 0.032
*wR*(*F* ^2^) = 0.119
*S* = 0.91
3208 reflections
307 parameters
H-atom parameters constrained
Δρ_max_ = 0.30 e Å^−3^
Δρ_min_ = −0.25 e Å^−3^

### 

Data collection: *COLLECT* (Nonius, 2001 ▶).; cell refinement: *DENZO* and *SCALEPACK* (Otwinowski & Minor, 1997 ▶); data reduction: *DENZO* and *SCALEPACK*; program(s) used to solve structure: *SIR92* (Altomare *et al.*, 1994 ▶); program(s) used to refine structure: *CRYSTALS* (Betteridge *et al.*, 2003 ▶); molecular graphics: *ORTEPII* (Johnson, 1976 ▶) in *TEXSAN* (Molecular Structure Corporation, 1997 ▶); software used to prepare material for publication: *CRYSTALS*.

## Supplementary Material

Crystal structure: contains datablocks I, global. DOI: 10.1107/S1600536809026889/sj2635sup1.cif

Structure factors: contains datablocks I. DOI: 10.1107/S1600536809026889/sj2635Isup2.hkl

Additional supplementary materials:  crystallographic information; 3D view; checkCIF report

## Figures and Tables

**Table 1:** Hydrogen-bond geometry (Å, °)

*D*—H⋯*A*	*D*—H	H⋯*A*	*D*⋯*A*	*D*—H⋯*A*
C17—H171⋯O4	0.97	2.33	3.046 (4)	130
C22—H222⋯O5^i^	0.97	2.43	3.374 (4)	165
C1—H11⋯O6	0.98	2.66	3.485 (4)	142
C10—H101⋯O6	0.97	2.70	3.477 (3)	138

Symmetry code: (i) 
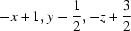
.

**Fig. 1.**
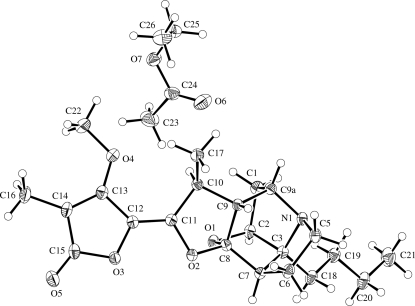
The molecular structure of stemofoline ethyl acetate solvate, with the atom labelling scheme. Displacement ellipsoids for non-H atoms are drawn at the 30% probability level.

**Fig. 2.**
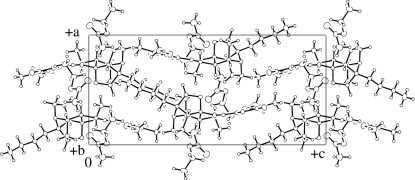
Unit-cell packing diagram of stemofoline ethyl acetate solvate.

## supplementary crystallographic information

### Comment

The crystallographic asymmetric unit consists of one C_22_H_29_NO_5_ molecule and one ethyl acetate molecule of crystallization. Examination of distances and angles around the N atom shows N4—C5 to be slightly shorter than the other two distances and for C5—N4—C9a to be significantly larger than the other two angles. These features were also observed in the closely related compounds reported by Seger *et al.* (2004). Unfortunately, coordinates are not available for the structure of stemofoline hydrobromide hydrate (Irie *et al.*, 1970).

### Experimental

Stemofoline was isolated from the root extracts of *Stemona aphylla* (Stemonaceae) as reported earlier (Mungkornasawakul *et al.*, 2009) and was crystallized from ethyl acetate/40–60°C petroleum ether. The sample had m.p. 71.5–73.2 °C (lit., Irie *et al.* (1970), 87–89 °C) and [α]_D_^23^ 269.1 (c 0.18, CHCl_3_) (lit., Irie *et al.* (1970), [α]_D_ 273 (MeOH)).

### Refinement

The compound is enantiometrically pure but the anomolous dispersion terms are very low for all elements in the structure and so the absolute configuration can not be determined in this experiment. Consequently Friedel-pair reflections have been averaged and the Flack parameter has not been refined. The molecule is presented with the same absolute configuration as that determined by X-ray crystallography for a closely related compound (Seger *et al.*, 2004). It is also consistent with that reported for stemofoline.HBr.H_2_O (Irie *et al.*, 1970), though the absolute structure determination performed there could possibly be regarded as not totally reliable.

All hydrogen atoms were observed in difference electron density maps prior to their inclusion in the structure. They were included at calculated positions and then were initially refined with soft restraints on the bond lengths and angles to regularize their geometry (C—H in the range 0.93–0.98 Å) and with *U*_iso_(H) in the range 1.2–1.5 times *U*_eq_ of the parent atom, after which the positions were refined with riding constraints.

One strong reflection with poor agreement between *F*_o_ and *F*_c_ was removed from the refinement as being unreliably measured.

The largest features in the final difference electron density map are located along C—C bonds.

### Crystal data

**Table:**

C_22_H_29_NO_5_·C_4_H_8_O_2_	*F*(000) = 1024
*M**_r_* = 475.58	*D*_x_ = 1.273 Mg m^−^^3^
Orthorhombic, *P*2_1_2_1_2_1_	Mo *K*α radiation, λ = 0.71073 Å
Hall symbol: P 2ac 2ab	Cell parameters from 36203 reflections
*a* = 10.3908 (1) Å	θ = 2.6–27.5°
*b* = 10.6549 (2) Å	µ = 0.09 mm^−^^1^
*c* = 22.4143 (4) Å	*T* = 200 K
*V* = 2481.55 (7) Å^3^	Needle, colourless
*Z* = 4	0.45 × 0.08 × 0.06 mm

### Data collection

**Table:**

Nonius KappaCCD diffractometer	2149 reflections with *I* > 2σ(*I*)
graphite	*R*_int_ = 0.060
φ and ω scans	θ_max_ = 27.5°, θ_min_ = 2.6°
Absorption correction: integration via Gaussian method (Coppens, 1970) implemented in *maXus* (Mackay *et al.*, 2000)	*h* = −11→13
*T*_min_ = 0.976, *T*_max_ = 0.996	*k* = −13→13
41745 measured reflections	*l* = −29→25
3209 independent reflections	

### Refinement

**Table:**

Refinement on *F*^2^	Primary atom site location: structure-invariant direct methods
Least-squares matrix: full	Hydrogen site location: inferred from neighbouring sites
*R*[*F*^2^ > 2σ(*F*^2^)] = 0.032	H-atom parameters constrained
*wR*(*F*^2^) = 0.119	Method, part 1, Chebychev polynomial, (Watkin, 1994, *P*rince, 1982) [*w*eight] = 1.0/[A_0_*T_0_(x) + A_1_*T_1_(x) ··· + A_n-1_]*T_n-1_(x)] where A_i_ are the Chebychev coefficients listed belo*w* and x = *F* /*F*max Method = Robust Weighting (*P*rince, 1982) W = [*w*eight] * [1-(delta*F*/6*sigma*F*)^2^]^2^ A_i_ are: 38.7 60.5 31.2 8.38
*S* = 0.91	(Δ/σ)_max_ = 0.002
3208 reflections	Δρ_max_ = 0.30 e Å^−^^3^
307 parameters	Δρ_min_ = −0.25 e Å^−^^3^
0 restraints	

### Fractional atomic coordinates and isotropic or equivalent isotropic displacement parameters (Å^2^)

**Table:**

	*x*	*y*	*z*	*U*_iso_*/*U*_eq_	
O1	0.43611 (16)	0.73200 (17)	0.47018 (7)	0.0301	
O2	0.27062 (18)	0.79684 (16)	0.52966 (7)	0.0295	
O3	0.30052 (17)	0.82685 (17)	0.64855 (8)	0.0315	
O4	0.3242 (2)	0.49666 (18)	0.65754 (9)	0.0430	
O5	0.3409 (2)	0.9075 (2)	0.73957 (9)	0.0469	
O6	0.4158 (3)	0.2904 (2)	0.50427 (12)	0.0612	
O7	0.4878 (2)	0.1608 (2)	0.57490 (9)	0.0427	
N4	0.25605 (19)	0.6497 (2)	0.35879 (8)	0.0278	
C1	0.4196 (3)	0.5479 (3)	0.40840 (11)	0.0331	
C2	0.4559 (2)	0.6861 (2)	0.41002 (10)	0.0302	
C3	0.3504 (2)	0.7525 (2)	0.37202 (10)	0.0287	
C5	0.1296 (2)	0.7117 (3)	0.35629 (11)	0.0344	
C6	0.1362 (3)	0.8327 (3)	0.39472 (11)	0.0355	
C7	0.2744 (2)	0.8344 (2)	0.41800 (10)	0.0286	
C8	0.2994 (2)	0.7520 (2)	0.47146 (10)	0.0265	
C9	0.2300 (2)	0.6269 (2)	0.46769 (10)	0.0257	
C9a	0.2729 (2)	0.5612 (2)	0.40984 (10)	0.0290	
C10	0.2550 (2)	0.5713 (2)	0.53031 (10)	0.0268	
C11	0.2737 (2)	0.6909 (2)	0.56580 (10)	0.0267	
C12	0.2931 (2)	0.7068 (2)	0.62431 (11)	0.0280	
C13	0.3204 (2)	0.6191 (3)	0.67198 (11)	0.0326	
C14	0.3440 (3)	0.6821 (3)	0.72309 (11)	0.0347	
C15	0.3298 (2)	0.8144 (3)	0.70898 (11)	0.0346	
C16	0.3825 (3)	0.6425 (3)	0.78503 (12)	0.0495	
C17	0.1423 (3)	0.4895 (3)	0.55107 (12)	0.0376	
C18	0.3966 (3)	0.8207 (3)	0.31649 (11)	0.0357	
C19	0.4653 (3)	0.7364 (3)	0.27164 (11)	0.0377	
C20	0.5063 (3)	0.8045 (3)	0.21512 (12)	0.0452	
C21	0.5627 (4)	0.7165 (4)	0.16876 (13)	0.0574	
C22	0.3592 (4)	0.4074 (3)	0.70266 (16)	0.0556	
C23	0.6093 (4)	0.3409 (4)	0.55692 (18)	0.0651	
C24	0.4940 (3)	0.2645 (3)	0.54151 (14)	0.0416	
C25	0.3810 (3)	0.0762 (3)	0.56316 (15)	0.0440	
C26	0.2641 (3)	0.1114 (3)	0.59907 (17)	0.0558	
H11	0.4519	0.5013	0.4432	0.0396*	
H12	0.4479	0.5081	0.3715	0.0395*	
H21	0.5450	0.7060	0.3962	0.0365*	
H911	0.2270	0.4811	0.4032	0.0338*	
H51	0.0633	0.6534	0.3702	0.0410*	
H52	0.1109	0.7355	0.3144	0.0413*	
H61	0.0745	0.8283	0.4278	0.0416*	
H62	0.1195	0.9067	0.3706	0.0420*	
H71	0.3101	0.9183	0.4223	0.0337*	
H91	0.1369	0.6445	0.4656	0.0306*	
H101	0.3345	0.5243	0.5313	0.0325*	
H161	0.3834	0.7165	0.8100	0.0738*	
H162	0.3207	0.5819	0.8013	0.0742*	
H163	0.4664	0.6037	0.7857	0.0738*	
H171	0.1554	0.4602	0.5914	0.0551*	
H172	0.0625	0.5382	0.5495	0.0547*	
H173	0.1341	0.4163	0.5249	0.0551*	
H181	0.4556	0.8879	0.3283	0.0420*	
H182	0.3199	0.8577	0.2961	0.0419*	
H191	0.5420	0.7010	0.2907	0.0449*	
H192	0.4080	0.6653	0.2615	0.0455*	
H201	0.5699	0.8696	0.2253	0.0540*	
H202	0.4287	0.8439	0.1984	0.0544*	
H211	0.5834	0.7639	0.1322	0.0857*	
H212	0.6415	0.6757	0.1828	0.0859*	
H213	0.4996	0.6521	0.1586	0.0860*	
H221	0.3518	0.3231	0.6844	0.0821*	
H222	0.4473	0.4219	0.7153	0.0819*	
H223	0.3003	0.4131	0.7367	0.0818*	
H231	0.6081	0.4193	0.5351	0.0959*	
H232	0.6114	0.3581	0.5997	0.0961*	
H233	0.6878	0.2943	0.5464	0.0963*	
H251	0.4091	−0.0102	0.5747	0.0533*	
H252	0.3601	0.0796	0.5197	0.0528*	
H261	0.1959	0.0507	0.5919	0.0841*	
H262	0.2843	0.1122	0.6421	0.0837*	
H263	0.2342	0.1963	0.5874	0.0839*	

### Atomic displacement parameters (Å^2^)

**Table:**

	*U*^11^	*U*^22^	*U*^33^	*U*^12^	*U*^13^	*U*^23^
O1	0.0236 (7)	0.0391 (9)	0.0275 (8)	−0.0033 (7)	−0.0031 (7)	0.0015 (7)
O2	0.0370 (8)	0.0293 (8)	0.0223 (7)	−0.0010 (7)	−0.0001 (7)	0.0000 (7)
O3	0.0339 (9)	0.0344 (9)	0.0263 (8)	−0.0039 (7)	−0.0009 (7)	−0.0017 (7)
O4	0.0571 (12)	0.0361 (10)	0.0359 (9)	0.0023 (9)	−0.0096 (9)	0.0058 (8)
O5	0.0530 (12)	0.0514 (12)	0.0363 (10)	−0.0041 (10)	−0.0027 (9)	−0.0099 (9)
O6	0.0657 (15)	0.0521 (14)	0.0658 (15)	0.0054 (12)	−0.0062 (13)	0.0190 (12)
O7	0.0420 (10)	0.0406 (10)	0.0456 (10)	−0.0051 (9)	−0.0037 (8)	0.0086 (9)
N4	0.0241 (9)	0.0348 (10)	0.0246 (9)	−0.0017 (8)	−0.0019 (7)	0.0005 (8)
C1	0.0330 (12)	0.0360 (13)	0.0304 (11)	0.0083 (10)	0.0025 (10)	0.0004 (10)
C2	0.0248 (11)	0.0404 (14)	0.0255 (10)	−0.0014 (10)	0.0004 (9)	0.0027 (10)
C3	0.0278 (11)	0.0322 (11)	0.0262 (10)	0.0006 (9)	−0.0008 (9)	0.0007 (10)
C5	0.0268 (11)	0.0450 (13)	0.0314 (11)	0.0020 (10)	−0.0069 (9)	−0.0007 (11)
C6	0.0355 (12)	0.0406 (13)	0.0303 (11)	0.0113 (11)	−0.0043 (10)	0.0025 (11)
C7	0.0318 (12)	0.0289 (11)	0.0253 (10)	−0.0009 (10)	−0.0016 (9)	0.0019 (10)
C8	0.0257 (10)	0.0286 (11)	0.0252 (10)	0.0002 (9)	−0.0017 (9)	−0.0030 (10)
C9	0.0238 (10)	0.0282 (11)	0.0250 (10)	−0.0008 (9)	−0.0009 (8)	0.0005 (9)
C9a	0.0304 (12)	0.0301 (11)	0.0265 (10)	−0.0016 (9)	0.0023 (9)	−0.0003 (9)
C10	0.0262 (10)	0.0275 (11)	0.0267 (10)	−0.0010 (9)	−0.0006 (9)	−0.0006 (9)
C11	0.0217 (10)	0.0307 (11)	0.0275 (10)	−0.0019 (9)	0.0012 (8)	0.0023 (9)
C12	0.0245 (10)	0.0326 (11)	0.0269 (11)	−0.0017 (9)	0.0009 (9)	−0.0013 (10)
C13	0.0274 (11)	0.0401 (13)	0.0304 (11)	−0.0012 (10)	−0.0006 (9)	0.0026 (10)
C14	0.0305 (12)	0.0492 (15)	0.0245 (10)	−0.0014 (11)	−0.0005 (9)	0.0041 (10)
C15	0.0278 (12)	0.0490 (15)	0.0271 (11)	−0.0034 (11)	−0.0020 (9)	−0.0010 (11)
C16	0.0594 (19)	0.0608 (19)	0.0281 (12)	−0.0049 (16)	−0.0091 (12)	0.0073 (13)
C17	0.0400 (14)	0.0379 (13)	0.0350 (12)	−0.0111 (11)	−0.0010 (11)	0.0053 (11)
C18	0.0366 (13)	0.0434 (14)	0.0270 (11)	−0.0039 (12)	0.0030 (10)	0.0071 (11)
C19	0.0356 (13)	0.0450 (15)	0.0325 (12)	−0.0036 (12)	0.0009 (10)	0.0062 (11)
C20	0.0440 (15)	0.0642 (18)	0.0275 (11)	−0.0026 (14)	0.0045 (11)	0.0076 (13)
C21	0.0618 (19)	0.076 (2)	0.0344 (14)	−0.0067 (18)	0.0077 (14)	−0.0012 (15)
C22	0.071 (2)	0.0446 (16)	0.0517 (17)	0.0004 (16)	−0.0158 (17)	0.0136 (14)
C23	0.073 (2)	0.0541 (19)	0.068 (2)	−0.0221 (19)	0.0059 (19)	−0.0021 (18)
C24	0.0468 (16)	0.0331 (13)	0.0451 (14)	0.0039 (12)	0.0067 (12)	−0.0018 (12)
C25	0.0415 (15)	0.0349 (13)	0.0558 (17)	−0.0041 (12)	−0.0001 (13)	0.0016 (13)
C26	0.0461 (17)	0.0518 (18)	0.070 (2)	−0.0022 (15)	0.0117 (16)	0.0055 (16)

### Geometric parameters (Å, °)

**Table:**

O1—C2	1.449 (3)	C10—H101	0.966
O1—C8	1.436 (3)	C11—C12	1.338 (3)
O2—C8	1.421 (3)	C12—C13	1.447 (3)
O2—C11	1.389 (3)	C13—C14	1.350 (4)
O3—C12	1.392 (3)	C14—C15	1.452 (4)
O3—C15	1.394 (3)	C14—C16	1.505 (3)
O4—C13	1.345 (3)	C16—H161	0.967
O4—C22	1.435 (3)	C16—H162	0.980
O5—C15	1.212 (3)	C16—H163	0.965
O6—C24	1.197 (4)	C17—H171	0.965
O7—C24	1.336 (4)	C17—H172	0.978
O7—C25	1.453 (3)	C17—H173	0.980
N4—C3	1.499 (3)	C18—C19	1.525 (4)
N4—C5	1.471 (3)	C18—H181	0.980
N4—C9a	1.493 (3)	C18—H182	1.000
C1—C2	1.520 (4)	C19—C20	1.521 (4)
C1—C9a	1.531 (3)	C19—H191	0.980
C1—H11	0.984	C19—H192	0.990
C1—H12	0.976	C20—C21	1.518 (5)
C2—C3	1.559 (3)	C20—H201	0.985
C2—H21	0.998	C20—H202	0.983
C3—C7	1.565 (3)	C21—H211	0.987
C3—C18	1.520 (3)	C21—H212	0.979
C5—C6	1.552 (4)	C21—H213	0.975
C5—H51	0.978	C22—H221	0.991
C5—H52	0.993	C22—H222	0.970
C6—C7	1.528 (3)	C22—H223	0.981
C6—H61	0.981	C23—C24	1.489 (5)
C6—H62	0.971	C23—H231	0.968
C7—C8	1.508 (3)	C23—H232	0.977
C7—H71	0.972	C23—H233	0.984
C8—C9	1.519 (3)	C25—C26	1.504 (4)
C9—C9a	1.539 (3)	C25—H251	1.000
C9—C10	1.545 (3)	C25—H252	0.999
C9—H91	0.986	C26—H261	0.973
C9a—H911	0.989	C26—H262	0.987
C10—C11	1.515 (3)	C26—H263	0.991
C10—C17	1.532 (3)		
			
O1···O2^i^	3.489 (2)	O5···C22^iv^	3.375 (5)
O2···C25^ii^	3.277 (4)	O6···C10	3.477 (3)
O2···C2^iii^	3.543 (3)	O6···C1	3.485 (4)
O3···C26^ii^	3.250 (4)	O7···C16^v^	3.422 (3)
O3···C25^ii^	3.379 (4)	C2···C11^i^	3.594 (3)
O3···C5^i^	3.446 (3)	C5···C15^iii^	3.453 (3)
			
C2—O1—C8	102.09 (17)	C11—C12—C13	132.2 (2)
C8—O2—C11	104.91 (18)	C12—C13—O4	117.0 (2)
C12—O3—C15	107.7 (2)	C12—C13—C14	109.9 (2)
C13—O4—C22	118.7 (2)	O4—C13—C14	133.0 (2)
C24—O7—C25	116.6 (2)	C13—C14—C15	106.2 (2)
C3—N4—C5	105.30 (19)	C13—C14—C16	133.7 (3)
C3—N4—C9a	103.48 (17)	C15—C14—C16	120.0 (2)
C5—N4—C9a	114.66 (18)	C14—C15—O3	109.1 (2)
C2—C1—C9a	99.0 (2)	C14—C15—O5	131.5 (2)
C2—C1—H11	112.7	O3—C15—O5	119.5 (3)
C9a—C1—H11	111.7	C14—C16—H161	107.8
C2—C1—H12	111.5	C14—C16—H162	110.6
C9a—C1—H12	111.0	H161—C16—H162	109.2
H11—C1—H12	110.5	C14—C16—H163	112.0
C1—C2—O1	108.29 (19)	H161—C16—H163	109.4
C1—C2—C3	104.6 (2)	H162—C16—H163	107.8
O1—C2—C3	104.78 (18)	C10—C17—H171	111.1
C1—C2—H21	115.4	C10—C17—H172	109.6
O1—C2—H21	110.4	H171—C17—H172	109.0
C3—C2—H21	112.8	C10—C17—H173	109.7
C2—C3—N4	103.71 (19)	H171—C17—H173	108.3
C2—C3—C7	104.38 (18)	H172—C17—H173	109.1
N4—C3—C7	102.02 (18)	C3—C18—C19	114.0 (2)
C2—C3—C18	116.2 (2)	C3—C18—H181	109.0
N4—C3—C18	113.22 (19)	C19—C18—H181	108.5
C7—C3—C18	115.6 (2)	C3—C18—H182	108.1
N4—C5—C6	108.20 (19)	C19—C18—H182	107.7
N4—C5—H51	109.4	H181—C18—H182	109.5
C6—C5—H51	112.4	C18—C19—C20	113.5 (2)
N4—C5—H52	109.0	C18—C19—H191	108.6
C6—C5—H52	108.7	C20—C19—H191	108.6
H51—C5—H52	109.1	C18—C19—H192	108.7
C5—C6—C7	103.9 (2)	C20—C19—H192	110.0
C5—C6—H61	110.5	H191—C19—H192	107.1
C7—C6—H61	110.9	C19—C20—C21	112.6 (3)
C5—C6—H62	111.0	C19—C20—H201	109.3
C7—C6—H62	110.3	C21—C20—H201	109.6
H61—C6—H62	110.0	C19—C20—H202	106.9
C6—C7—C3	104.02 (19)	C21—C20—H202	108.7
C6—C7—C8	115.2 (2)	H201—C20—H202	109.7
C3—C7—C8	96.39 (18)	C20—C21—H211	109.6
C6—C7—H71	113.8	C20—C21—H212	112.1
C3—C7—H71	112.7	H211—C21—H212	108.2
C8—C7—H71	113.0	C20—C21—H213	109.6
C7—C8—O1	103.93 (19)	H211—C21—H213	108.2
C7—C8—O2	119.8 (2)	H212—C21—H213	108.9
O1—C8—O2	106.06 (17)	O4—C22—H221	106.9
C7—C8—C9	112.66 (18)	O4—C22—H222	109.8
O1—C8—C9	109.78 (19)	H221—C22—H222	109.7
O2—C8—C9	104.25 (18)	O4—C22—H223	110.5
C8—C9—C9a	107.95 (18)	H221—C22—H223	109.2
C8—C9—C10	101.88 (18)	H222—C22—H223	110.6
C9a—C9—C10	122.82 (19)	C24—C23—H231	110.1
C8—C9—H91	107.6	C24—C23—H232	110.4
C9a—C9—H91	109.3	H231—C23—H232	109.6
C10—C9—H91	106.4	C24—C23—H233	109.6
C9—C9a—C1	110.4 (2)	H231—C23—H233	108.9
C9—C9a—N4	108.94 (19)	H232—C23—H233	108.1
C1—C9a—N4	99.16 (19)	C23—C24—O7	111.2 (3)
C9—C9a—H911	112.3	C23—C24—O6	125.6 (3)
C1—C9a—H911	113.4	O7—C24—O6	123.3 (3)
N4—C9a—H911	111.9	O7—C25—C26	111.4 (3)
C9—C10—C11	100.13 (17)	O7—C25—H251	107.5
C9—C10—C17	111.4 (2)	C26—C25—H251	109.1
C11—C10—C17	114.6 (2)	O7—C25—H252	108.7
C9—C10—H101	111.4	C26—C25—H252	109.7
C11—C10—H101	108.3	H251—C25—H252	110.3
C17—C10—H101	110.6	C25—C26—H261	109.6
C10—C11—O2	111.99 (19)	C25—C26—H262	110.7
C10—C11—C12	129.8 (2)	H261—C26—H262	108.8
O2—C11—C12	118.2 (2)	C25—C26—H263	109.8
O3—C12—C11	120.5 (2)	H261—C26—H263	109.5
O3—C12—C13	107.1 (2)	H262—C26—H263	108.4
			
O1—C2—C1—C9a	75.1 (2)	C2—C3—N4—C5	147.5 (2)
O1—C2—C3—N4	−107.2 (2)	C2—C3—N4—C9a	26.8 (2)
O1—C2—C3—C7	−0.7 (2)	C2—C3—C7—C6	−146.3 (2)
O1—C2—C3—C18	127.9 (2)	C2—C3—C7—C8	−28.3 (2)
O1—C8—O2—C11	−77.4 (2)	C2—C3—C18—C19	59.5 (3)
O1—C8—C7—C3	49.8 (2)	C3—N4—C5—C6	−25.4 (2)
O1—C8—C7—C6	158.7 (2)	C3—N4—C9a—C9	65.3 (2)
O1—C8—C9—C10	72.6 (2)	C3—C2—O1—C8	31.6 (2)
O1—C8—C9—C9a	−57.9 (2)	C3—C2—C1—C9a	−36.3 (2)
O2—C8—O1—C2	179.6 (2)	C3—C7—C6—C5	23.2 (2)
O2—C8—C7—C3	167.9 (2)	C3—C7—C8—C9	−68.9 (2)
O2—C8—C7—C6	−83.2 (3)	C3—C18—C19—C20	177.6 (2)
O2—C8—C9—C10	−40.6 (2)	C5—N4—C3—C7	39.3 (2)
O2—C8—C9—C9a	−171.2 (2)	C5—N4—C3—C18	−85.6 (2)
O2—C11—C10—C9	−4.1 (2)	C5—N4—C9a—C9	−48.8 (2)
O2—C11—C10—C17	−123.5 (2)	C5—C6—C7—C8	−81.0 (2)
O2—C11—C12—O3	4.6 (3)	C6—C5—N4—C9a	87.6 (2)
O2—C11—C12—C13	−169.4 (2)	C6—C7—C3—C18	84.6 (2)
O3—C12—C11—C10	−176.2 (2)	C6—C7—C8—C9	39.9 (3)
O3—C12—C13—O4	−177.0 (2)	C7—C3—N4—C9a	−81.4 (2)
O3—C12—C13—C14	−0.2 (3)	C7—C3—C18—C19	−177.6 (2)
O3—C15—C14—C13	−1.4 (3)	C7—C8—O2—C11	165.6 (2)
O3—C15—C14—C16	176.9 (2)	C7—C8—C9—C10	−172.1 (2)
O4—C13—C12—C11	−2.4 (4)	C7—C8—C9—C9a	57.4 (2)
O4—C13—C14—C15	177.0 (2)	C8—O2—C11—C10	−21.4 (2)
O4—C13—C14—C16	−0.9 (5)	C8—O2—C11—C12	157.9 (2)
O5—C15—O3—C12	−179.8 (2)	C8—C7—C3—C18	−157.3 (2)
O5—C15—C14—C13	179.9 (3)	C8—C9—C10—C11	26.1 (2)
O5—C15—C14—C16	−1.9 (4)	C8—C9—C10—C17	147.8 (2)
O6—C24—O7—C25	1.4 (4)	C9—C8—O2—C11	38.5 (2)
N4—C3—C2—C1	6.7 (2)	C9—C10—C11—C12	176.6 (2)
N4—C3—C7—C6	−38.7 (2)	C10—C11—C12—C13	9.8 (4)
N4—C3—C7—C8	79.4 (2)	C11—C10—C9—C9a	146.8 (2)
N4—C3—C18—C19	−60.4 (3)	C11—C12—O3—C15	−176.0 (2)
N4—C5—C6—C7	0.6 (3)	C11—C12—C13—C14	174.4 (3)
N4—C9a—C1—C2	52.8 (2)	C12—O3—C15—C14	1.2 (2)
N4—C9a—C9—C8	−51.8 (2)	C12—C11—C10—C17	57.2 (3)
N4—C9a—C9—C10	−169.7 (2)	C12—C13—O4—C22	176.0 (2)
C1—C2—O1—C8	−79.7 (2)	C12—C13—C14—C15	0.9 (3)
C1—C2—C3—C7	113.1 (2)	C12—C13—C14—C16	−177.0 (3)
C1—C2—C3—C18	−118.2 (2)	C13—C12—O3—C15	−0.6 (2)
C1—C9a—N4—C3	−50.1 (2)	C14—C13—O4—C22	0.1 (4)
C1—C9a—N4—C5	−164.2 (2)	C17—C10—C9—C9a	−91.5 (2)
C1—C9a—C9—C8	56.1 (2)	C18—C3—N4—C9a	153.7 (2)
C1—C9a—C9—C10	−61.8 (3)	C18—C19—C20—C21	−174.5 (3)
C2—O1—C8—C7	−53.2 (2)	C23—C24—O7—C25	−178.5 (3)
C2—O1—C8—C9	67.5 (2)	C24—O7—C25—C26	−87.9 (3)
C2—C1—C9a—C9	−61.4 (2)		

Symmetry codes: (i) *x*+1/2, −*y*+3/2, −*z*+1; (ii) *x*, *y*+1, *z*; (iii) *x*−1/2, −*y*+3/2, −*z*+1; (iv) −*x*+1, *y*+1/2, −*z*+3/2; (v) −*x*+1, *y*−1/2, −*z*+3/2.

### Hydrogen-bond geometry (Å, °)

**Table:**

*D*—H···*A*	*D*—H	H···*A*	*D*···*A*	*D*—H···*A*
C17—H171···O4	0.97	2.33	3.046 (4)	130
C22—H222···O5^v^	0.97	2.43	3.374 (4)	165
C1—H11···O6	0.98	2.66	3.485 (4)	142
C10—H101···O6	0.97	2.70	3.477 (3)	138

Symmetry codes: (v) −*x*+1, *y*−1/2, −*z*+3/2.
